# Validity and reliability of a brief self-reported questionnaire assessing fruit and vegetable consumption among pregnant women

**DOI:** 10.1186/s12889-016-3656-y

**Published:** 2016-09-15

**Authors:** Lydi-Anne Vézina-Im, Gaston Godin, Charles Couillard, Julie Perron, Simone Lemieux, Julie Robitaille

**Affiliations:** 1 School of Nutrition, Laval University, Quebec City, QC G1V 0A6 Canada; 2 Institute of Nutrition and Functional Foods, Laval University, Quebec City, QC Canada; 3 Faculty of Nursing, Laval University, Quebec City, QC Canada; 4 Endocrinology and Nephrology Axis, CHU de Quebec Research Center, Quebec City, QC Canada

**Keywords:** Fruit and vegetable, Pregnancy, Questionnaire, Validity, Reliability

## Abstract

**Background:**

Short instruments measuring frequency of specific foods, such as fruit and vegetable (FV), are increasingly used in interventions. The objective of the study was to verify the validity and test-retest reliability of such an instrument among pregnant women.

**Methods:**

Pregnant women from the region of Quebec City, Quebec, Canada, were recruited through e-mails sent to female students and employees of the local university from October 2014 to April 2015. To assess the validity of the fruit and vegetable questionnaire (FVQ) developed by Godin et al. (Can J Public Health 99: 494-498, 2008), pregnant women were asked in a first mailing to complete the FVQ assessing FV intake over the past 7 days and a 3-day estimated food record. A subsample (*n* = 33) also gave a fasting blood sample and completed a validated semi-quantitative FFQ administered by a trained registered dietitian during a visit at the research center. FV intakes for all instruments were calculated in terms of servings of FV based on Canada’s Food Guide definition of a serving of fruit or vegetable. In order to assess its test-retest reliability, respondents were asked to complete the FVQ 14 days later in a second mailing.

**Results:**

Forty-eight pregnant women from all three trimesters completed the questionnaires in the first mailing. FV intake assessed using the FVQ was correlated to FV consumption measured using the food record (*r* = 0.34, *p* = 0.0180) and the FFQ (*r* = 0.61, *p* = 0.0002). Results were similar when controlling for energy intake and the experience of nausea in the past month. Only β-cryptoxanthin was significantly correlated to FV intake assessed by the FFQ when adjusted for the presence of nausea (*r* = 0.35, *p* = 0.0471). Data on the test-retest reliability was available for 44 women and the intra-class coefficient for the FVQ was 0.72 at a mean 28-day interval.

**Conclusions:**

The FVQ has acceptable validity and test-retest reliability values, but seems to underestimate FV servings in pregnant women. It represents an interesting alternative for researchers or clinicians interested in estimating quickly FV intake among pregnant women, such as in large trials or during prenatal visits. The FVQ should however be coupled with other self-reported measures, such as a food record, for assessing precise individual FV intake.

**Electronic supplementary material:**

The online version of this article (doi:10.1186/s12889-016-3656-y) contains supplementary material, which is available to authorized users.

## Background

Eating fruit and vegetables (FV) has numerous health benefits. Their consumption can lower risks of coronary heart diseases [1, 2], such as stroke [3, 4] and type 2 diabetes [5]. FV intake can also prevent different types of cancer, such as pancreatic cancer [6], gastric cancer [7], colorectal cancer [8] and breast cancer [9, 10].

Among pregnant women, a healthy diet comprised of FV is associated with a reduction in the risk of developing gestational diabetes mellitus (GDM) [11]. GDM is a glucose intolerance that first appears or is diagnosed during pregnancy that can lead to serious health consequences, such as high risks of developing future type 2 diabetes in mothers and their child [12–14]. After pregnancy, women with prior GDM are also at risks of developing cardiovascular diseases [15] and renal problems [16]. Children exposed *in-utero* to GDM have greater odds of becoming obese during childhood [17], regardless of their mother’s body mass index (BMI) [18]. Despite the health benefits, only between 35 and 54 % of pregnant women have FV intakes that meet public health recommendations in the UK and in Canada [19, 20].

In nutritional interventions, short instruments measuring frequency of foods targeted in the intervention, such as FV, are increasingly being used [21]. One example of such a tool is a brief one-page questionnaire developed by Godin et al. [22] that measures frequency of FV intake in servings over the past seven days. This fruit and vegetable questionnaire (FVQ) was significantly correlated to a validated interviewer-administered FFQ [23] in a sample of 350 obese (*r* = 0.66, *p* < 0.000) and non-obese individuals (*r* = 0.65, *p* < 0.0001) [22].

However, to our knowledge, this tool has never been validated in pregnant women and evidence of its test-retest reliability—which implies administering a same instrument to the same individuals on two different occasions [24]—has not been reported. A recent systematic review of self-reported measures of foods and nutrients in pregnancy highlighted the need to validate such instruments in pregnant women, since pregnancy involves an increase in nutritional needs and sometimes the experience of nausea [25]. Moreover, that same review stressed the need to report information on both the validity and reliability of self-reported tools, given that both are needed to assess their psychometric properties. For example, a researcher could mistakenly think that a woman’s diet changed over the course of her pregnancy while this is merely an artifact of a tool with poor reproducibility [25]. The objective of the present study was thus to evaluate the FVQ in a sample of pregnant women by estimating its validity and test-retest reliability.

## Methods

### Population and sample

Pregnant women from the region of Quebec City, Quebec, Canada, were recruited through e-mails sent to female students and employees of the local university from October 2014 to April 2015. The only inclusion criterion was to be pregnant. The study was reviewed and approved by the Research Ethics Committee of the Centre hospitalier universitaire (CHU) de Quebec and all women gave their informed consent prior to their inclusion in the study.

### Data collection

*Validity*. The validity of the FVQ was verified by comparing FV servings with those from a 3-day estimated food record, a validated semi-quantitative FFQ administered by a trained registered dietitian [23] and an objective measure of FV intake, plasma carotenoids concentrations. A food record and a FFQ were used as reference and comparison methods, respectively, given that a previous systematic review of self-reported measures of foods and nutrients in pregnancy found that FFQ and food records have the strongest evidence of validity in assessing nutrition during pregnancy [25]. The food record chosen was selected because it was developed for a French-Canadian population (i.e., available in French). For comparison purposes, the FFQ chosen was the same that was originally used in Godin et al.’s [22] study among obese and non-obese individuals. This will allow comparison of the correlations among pregnant women with those obtained in the original validation study. Plasma carotenoids concentrations were chosen given that they can be used as biomarkers of FV consumption [26–28], including among pregnant women [29]. Carotenoids included retinol, α-tocopherol, lutein, zeaxanthin, β-cryptoxanthin, α-carotene, β-carotene, lycopene (5-cis-lycopene, 9-cis-lycopene, 13-cis-lycopene and trans-lycopene) and total carotenoids. Higher concentrations of plasma carotenoids are expected in people who eat more servings of FV and certain fruit and vegetables are more correlated to specific carotenoids. For example, fruit intake is more strongly correlated to β-cryptoxanthin concentrations, tomatoes to lycopene and α-carotene to carrots [26].

*Procedure*. Pregnant women received two mailings. The first mailing contained a cover letter, an informed consent form, the FVQ, a food record and a pre-paid pre-addressed return envelope. In the cover letter, pregnant women were told to carefully read instructions on how to complete the FVQ and the food record and to indicate the date of completion. The instructions on how to complete the FVQ were given before those on how to complete the food record, but pregnant women were not specifically instructed in which order to fill out the FVQ and the food record. To assess reproducibility of the FVQ, a second mailing, containing only the FVQ and a pre-paid pre-addressed envelope, was sent approximately 14 days after the date at which the first FVQ was filled.

*Questionnaires and Biomarkers*. At the beginning of the FVQ, a definition of a serving of fruit or vegetable based on Canada’s Food Guide is provided (see Additional file 1 for the complete questionnaire). For example, one serving of fruit or vegetable is the equivalent of half a cup (125 ml) of cut fruits or vegetables [30]. The FVQ contains 3 questions on FV intake. For the first question, participants have to write down how many servings of FV divided into fruit juice, vegetable juice, potatoes (excluding French-fried potatoes), green salad and other vegetables they have consumed in the past seven days. For the second question, they have to indicate whether their FV consumption of the past seven days reflects their eating habits of the past three months. For the third question, they have to indicate how their eating habits of the past seven days differ from those of the past three months (e.g., much/a bit more/less FV than in the past three months). This was followed by a series of questions to collect sociodemographic data. For example, there were questions on age, the use of supplements and the experience of nausea in the past month in order to adjust for these factors in the statistical analyses. Unfortunately, we were unable to adjust for the use of supplements as only two pregnant women reported not using supplements, mainly prenatal multivitamins with folic acid. Completion of the FVQ took on average between 10 and 15 min.

For the food record, women were instructed to write down everything they ate and drank for two weekdays and one weekend day. At the end of the document, pregnant women had a space where they could write down recipes of foods they ate. Pregnant women were asked to measure the amount of food consumed with household measurements (e.g., cups, teaspoons, tablespoons) or to write down the quantity written on commercial packaging.

Upon entry into the study, pregnant women were also asked whether they would be interested in an optional part of the study which required a visit at the research center to give a blood sample and fill out the FFQ. During that visit, which usually occurred within two weeks after filling the FVQ and the food record, a fasting blood sample was drawn and dietary intake was assessed using a FFQ administered by a trained registered dietitian [23]. Before the visit, pregnant women were instructed to fast for at least 12 h before their scheduled blood sample (only water was allowed), to avoid drinking alcohol the previous 48 h and also to avoid doing intense physical activity the night before their appointment. A blood sample of 12 ml was collected by a venipuncture on their non-dominant arm from a trained nurse. Immediately after being collected, blood samples were stored in a refrigerator kept at a temperature of -80 °C until the assessment of plasma carotenoids concentrations. The FFQ inquired about eating habits of the past month and included 91 items that were listed in food groups (vegetables, fruit, legumes, nuts and seeds, cereals and grain products, milk and dairy products, meat and processed meat, poultry, fish, eggs, sweets, oils and fats, fast foods and drinks). Five items were concerning FV intake (vegetables, potatoes, vegetable or tomato juice, fruit and fruit juice). Visits lasted on average one hour.

When computing the number of FV portions, both the food record and the FFQ used the same algorithm which included FV consumed alone, dried (i.e., dried fruits) and also those contained in popular mixed dished. FV intakes for the FVQ, the food record and the FFQ were calculated in terms of servings of FV based on Canada’s Food Guide definition of a serving of fruit or vegetable [30]. Evaluation of energy intake from food records and FFQ was performed using the Nutrition Data System for Research (NDS-R) version 4.03, developed by the Nutrition Coordinating Center, University of Minnesota, Minneapolis, MN, Food and Nutrient Database 31.

Retinyl acetate, α-carotene, lutein, zeaxanthin, β-cryptoxanthin, β-carotene and lycopene were purchased from Sigma (Oakville, Ontario, Canada). All solvents were of high-performance liquid chromatography (HPLC) grade and purchased from VWR (Mississauga, Ontario, Canada). HPLC water was obtained using a MilliQ water purification system from Millipore (Etobicoke, Ontario, Canada). Stock solutions for each carotenoid were prepared (1 mg in 100 mL of solvent) in either ethanol (C2H6O; for lutein, zeaxanthin and β-cryptoxanthin) or hexane (C6H14; for β-carotene and lycopene). Solutions were left to shake overnight at 4 °C under dim light. The exact concentration of each stock solution was then determined using a UV spectrophotometer and the specific molecular extinction coefficient (e) of each carotenoid [31]. Appropriate volumes of stock solutions were then transferred to amber Eppendorf tubes and evaporated under nitrogen. On the day of the analyses, carotenoid standards were solubilized with methanol/dichloromethane (65/35, v/v) to obtain a final concentration of 2 mM. These solutions were then diluted to perform calibration curves. Retinyl acetate (15 mM) was used as an internal standard.

Post-intervention plasma samples kept at -80 °C were thawed a day before analysis. Samples were vortexed and then centrifuged at 3500 rpm for 10 min at 4 °C. Aliquots of 100 μL of plasma were then transferred in Eppendorf tubes (1.5 mL) along with 20 μL of 2-propanol and 20 μL of carotenoid standard and the tubes were vortexed. Samples were transferred on a 400 μL fixed well plate (ISOLUTE® SLE+, Biotage, Charlotte, NC) and 900 μL of hexane:isopropanol (90/10, v/v) was added to each well. Each extracted sample was evaporated under nitrogen and once dried, was reconstituted with 300 μL of methanol:dichloromethane (65/35, v/v). Plates were shaken for 10 min and samples were transferred into HPLC glass vials to be analyzed.

HPLC-UV analysis of the samples was performed using an Agilent 1260 liquid handling system (Agilent, Mississauga, Ontario, Canada) equipped with a binary pump system and a C30 reversed phase column (YMC America Inc., Allentown, PA) kept at constant temperature (35 °C). Carotenoids of the different samples were separated with a mobile phase consisting of methanol:water (98/2, v/v; Eluent A) and methyl-tert-butyl ether (MTBE; Eluent B; VWR, Mississauga, Ontario, Canada). Flow-rate was set at 1 mL/min and the gradient elution was as follows: 2 % Eluent B (initial), 2.0-80 % Eluent B (0.0-27.0 min), isocratic 80 % Eluent B (27.0-31.0 min), 80.0-2.0 % Eluent B (31.0-31.1 min) and isocratic 2 % Eluent B (31.1-34.0 min). UV detector was set at 450 nm and identification of each compound was confirmed using retention time and UV spectra (190-640 nm) of the pure compounds. Data acquisition was carried out with the Chemstation software (Agilent, Mississauga, Ontario, Canada).

### Statistical analyses

Descriptive statistics were used to describe the sample at baseline.

*Validity*. Mean servings of FV were compared using paired *t*-test analyses for the FVQ and the food record, the reference method. The proportion of pregnant women who met Canada’s Food Guide recommendation of at least seven servings of FV per day was compared using McNemar’s [32] test for the FVQ and the food record. The capacity of the FVQ to correctly classify pregnant women according to Canada’s Food Guide recommendation for FV was verified by means of standard epidemiologic indices (specificity, sensitivity, predictive values and accuracy) [33, 34]. The food record was used as the measure of "true" FV intake. Sensitivity represents the proportion of pregnant women who "truly" ate at least 7 servings of FV per day who were correctly classified as such by the FVQ. Specificity is the proportion of pregnant women who "truly" ate *less* than 7 servings of FV per day who were correctly classified as such by the FVQ. Predictive values contain the positive predictive value and the negative predictive value. The positive predictive value is the proportion of pregnant women classified as eating at least 7 servings of FV per day among pregnant women who "truly" ate at least 7 servings of FV per day. The negative predictive value is the proportion of pregnant women classified as eating *less* than 7 servings of FV per day among pregnant women who "truly" ate *less* than 7 servings of FV per day. The accuracy or correct classification rate is the proportion of classifications for which the FVQ and the food record agree.

Normality of the distribution of the variables was verified using Shapiro-Wilk’s test and both Pearson and Spearman correlations were computed for each variable. Both correlations were similar for all variables and therefore only the Pearson correlations are presented. Pearson and partial correlations (adjusted for energy intake and experience of nausea in the past month) were computed between the FVQ and the two reference methods (food record and plasma carotenoids concentrations) and with the comparison method (FFQ). The mean daily energy intake for each pregnant woman was derived from either their food record or the FFQ. Correlations were classified according to Cohen’s [35] criteria whereby correlations of 0.10 indicate a small effect size, 0.30 indicate a medium effect size and 0.50 indicate a large effect size. Finally, a Bland-Altman plot [36] was drawn to measure agreement between FV intakes measured by the FVQ and the food record.

*Test-retest Reliability*. The FVQ’s test-retest reliability was verified by computing intra-class correlations (ICCs) between the first and the second assessments of FV intakes. ICCs were classified according to Fermanian’s [37] criteria whereby ICCs between 0–0.30 are considered very bad or null, 0.31–0.50 are considered mediocre, 0.51–0.70 are considered moderate, 0.71–0.90 are considered good and over 0.91 are considered very good. All statistical analyses were computed using SAS version 9.4 (SAS Institute, Cary, NC, USA).

## Results

### Flow of participants and sample characteristics

**Fig. 1:**
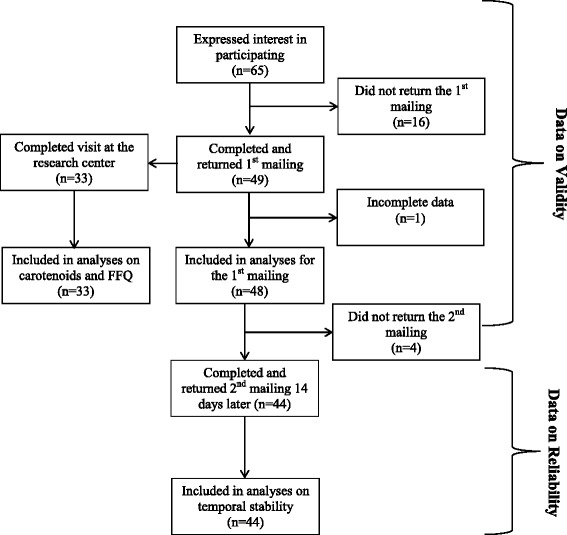
Flow of Participants

Sixty-five pregnant women expressed interest in participating in the study and 49 (response rate: 75.4 %) completed and returned the first mailing (see Fig. 1 for the complete flow of participants). One participant was removed from the statistical analyses as she reported having consumed zero FV in the past seven days while she reported high intakes of fresh FV in both the food record and the FFQ and it was assumed that it was a misunderstanding on how to complete the FVQ. Thus, the data from 48 pregnant women were analyzed for the first mailing. In addition, 33 respondents (participation rate: 67.4 %) participated in the optional part of the study that is, the visit at the research center where a fasting blood sample was drawn and the FFQ was administered by a trained registered dietitian. At the second mailing, 44 pregnant women (attrition rate: 10.2 %) completed and returned the FVQ. At baseline, the four pregnant women who did not complete the second mailing were similar to the other women in terms of age (32.5 ± 3.3 years), gestational weeks (24.5 ± 7.3 weeks), pre-pregnancy BMI (22.7 ± 2.8 kg/m^2^), level of education (75 % had a university degree) and parity (75 % were pregnant with their first child). They also had a high annual family income (> CA$ 100 000), none of them had a medical condition, all of them reported taking supplements and none of them reported having experienced nausea in the past month. The complete baseline characteristics of participants are presented in Table 1.

**Table 1:** Baseline characteristics of participants (*n* = 48)

Variables	Mean or %	SD
Age (years)	31.0	4.1
Gestational weeks	19.1	7.6
1^st^ trimester (0–14 weeks)	25.0 %	
2^nd^ trimester (15–28 weeks)	60.4 %	
3^rd^ trimester (29–42 weeks)	14.6 %	
Pre-pregnancy BMI	23.4	3.3
Normal weight (18.5–24.9 kg/m^2^)	72.9 %	
Overweight/obese (≥25 kg/m^2^)	27.1 %	
Education		
< university degree	14.6 %	
University degree	85.4 %	
Family annual income (CA$)		
0–100 000	56.3 %	
> 100 000	43.7 %	
Number of children		
0^a^	58.3 %	
1	22.9 %	
≥ 2	18.8 %	
Type of pregnancy		
Singleton	100 %	
Multiple	0 %	
Pregnancy with a medical condition^b^		
Yes	2.1 %	
No	97.9 %	
Supplements use		
Yes	95.8 %	
No	4.2 %	
Experienced nausea in the past month		
Yes	45.8 %	
No	54.2 %	
Nausea affected diet^c^		
Yes	68.2 %	
No	31.8 %	

*Abbreviations: SD* standard deviation, *BMI* body mass index

^a^Pregnant with their first child; ^b^Medical conditions included hypothyroidism and unicornuate uterus; ^c^Percentages only among the pregnant women who reported having experienced nausea in the past month

### Validity

The total number of servings for FV between the FVQ and the food record was significantly different (*p* = 0.0130). Yet, the differences in servings of vegetables, potatoes, and fruits were all non-significant (all *p*s > 0.05), except for vegetable juice (*p* = 0.0168) and fruit juice (*p* = 0.0134) (see Table 2). The percentages of pregnant women who followed or not the recommendations were not statistically different between the FVQ and the food record (*p* = 0.0736). When pregnant women were classified as eating at least 7 servings of FV per day or not, the sensitivity for the FVQ was 53.3 % while its specificity was 66.7 %. The positive predictive value was 72.7 % and the negative predictive value was 46.2 %. Finally, the accuracy of the FVQ to classify pregnant women as meeting or not Canada’s Food Guide recommendations for FV compared to the food record was 58.3 %.

**Table 2:** Comparison of the mean fruit and vegetable intakes and percentages of women who met Canada’s food guide recommendations for the fruit and vegetable questionnaire and the food record (*n* = 48)

Variables	FVQ	Food record	P-values
*Mean ± SD for daily intakes* (*servings*)			
Vegetables	2.53 ± 0.97	2.74 ± 1.41	0.2509
Potatoes^a^	0.29 ± 0.27	0.43 ± 1.02	0.3628
Vegetable juice	0.21 ± 0.39	0.44 ± 0.67	0.0168
Fruits	2.35 ± 0.88	2.48 ± 1.05	0.4390
Fruit juice	1.11 ± 0.81	1.50 ± 1.25	0.0134
Total fruit and vegetables	6.50 ± 1.78	7.41 ± 2.40	0.0130
*Canada’s Food Guide recommendations*			
< 7 servings of fruits and vegetables	54.2 %	37.5 %	0.0736
≥ 7 servings of fruits and vegetables	45.8 %	62.5 %	0.0736

*Abbreviations: FVQ* fruit and vegetable questionnaire, *SD* standard deviation

^a^Excluding French-fried potatoes

The FVQ was significantly correlated to the food record for all FV (range: 0.30–0.56, all *p*s < 0.05), except for potatoes (*r* = 0.08, *p* > 0.05) (see Table 3). The correlation for FV intakes between the FVQ and the food record was 0.34 (*p* = 0.0180). The correlations were similar when adjusted for energy intake and the experience of nausea in the past month. The majority of the correlations (crude and adjusted for energy and for nausea) between the FVQ and the food record represented medium-to-large effect sizes (*r* between 0.30 and 0.50).

**Table 3:** Correlations between the fruit and vegetable questionnaire, the food record and the food-frequency questionnaire

	Crude Pearson Correlations	Partial Correlations Adjusted for Energy Intake	Partial Correlations Adjusted for Nausea	Partial Correlations Adjusted for Energy Intake and Nausea
FVQ vs. food record (*n* = 48)				
Vegetables	0.46**	0.45**	0.44**	0.43**
Potatoes^a^	0.08	0.04	0.07	0.03
Vegetable juice	0.38**	0.39**	0.38**	0.39**
Fruits	0.30*	0.33*	0.29*	0.32*
Fruit juice	0.56***	0.57***	0.56***	0.56***
Fruits and vegetables	0.34*	0.33*	0.33*	0.32*
FVQ vs. FFQ (*n* = 33)				
Vegetables	0.70***	0.72***	0.69***	0.72***
Potatoes^a^	0.19	0.19	0.21	0.20
Vegetable juice	0.76***	0.75***	0.76***	0.76***
Fruits	0.72***	0.72***	0.73***	0.73***
Fruit juice	0.73***	0.71***	0.74***	0.72***
Fruits and vegetables	0.61**	0.57**	0.60**	0.55**

*Abbreviations: FVQ* fruit and vegetable questionnaire, *FFQ* food-frequency questionnaire

*p < 0.05 **p < 0.01 ***p < 0.0001

^a^Excluding French-fried potatoes

The FVQ was also significantly correlated to the FFQ for all FV (range: 0.61–0.76, all *p*s < 0.05), except for potatoes (*r* = 0.19, *p* > 0.05) (see Table 3). The correlation for FV intakes between the FVQ and the FFQ was 0.61 (*p* = 0.0002). The correlations were similar after adjustments for energy intake and for experience of nausea^1^ in the past months. The correlations (crude and adjusted for energy and for nausea) between the FVQ and the FFQ represented mainly large effect sizes (*r* ≥ 0.50).

FV intake, excluding potatoes, assessed using the FVQ, the food record or the FFQ was not significantly correlated to any of the plasma carotenoids assessed or to total plasma carotenoids concentrations (range: -0.29–0.30, all *p*s > 0.05) (see Table 4). Adjustments for energy intake and the experience of nausea^1^ in the past month did not modify the correlations, except that the correlation between the FFQ and β-cryptoxanthin plasma concentrations became significant (*r* = 0.35, *p* = 0.0471) and represents a medium effect size.

**Table 4:** Correlations between fruit and vegetable^a^ intake and plasma carotenoids concentrations

	Crude Pearson Correlations	Partial Correlations Adjusted for Energy Intake	Partial Correlations Adjusted for Nausea	Partial Correlations Adjusted for Energy Intake and Nausea^b^
FVQ (*n* = 48)				
Retinol	0.21	N/A	0.23	N/A
α-tocopherol	−0.17	N/A	−0.06	N/A
Lutein	−0.08	N/A	−0.07	N/A
Zeaxanthin	−0.16	N/A	−0.11	N/A
β-cryptoxanthin	0.02	N/A	0.04	N/A
α-carotene	0.11	N/A	0.13	N/A
β-carotene	0.05	N/A	0.10	N/A
Lycopene	−0.27	N/A	−0.23	N/A
Total carotenoids	−0.16	N/A	−0.05	N/A
Food record (*n* = 48)				
Retinol	0.04	−0.06	0.04	−0.05
α-tocopherol	−0.21	−0.24	−0.19	−0.23
Lutein	0.15	0.15	0.15	0.15
Zeaxanthin	0.01	−0.06	0.03	−0.04
β-cryptoxanthin	0.07	−0.02	0.07	−0.02
α-carotene	0.07	0.02	0.08	0.02
β-carotene	−0.08	−0.11	−0.06	−0.10
Lycopene	−0.11	−0.22	−0.09	−0.20
Total carotenoids	−0.20	−0.24	−0.18	−0.23
FFQ (*n* = 33)				
Retinol	0.30	0.31	0.31	0.32
α-tocopherol	−0.06	−0.05	0.01	0.02
Lutein	0.24	0.24	0.24	0.25
Zeaxanthin	0.18	0.17	0.22	0.21
β-cryptoxanthin	0.34	0.31	0.35*	0.32
α-carotene	0.19	0.21	0.21	0.22
β-carotene	0.19	0.16	0.22	0.19
Lycopene	0.08	0.13	0.11	0.17
Total carotenoids	−0.02	−0.01	0.05	0.07

*Abbreviations: FVQ* fruit and vegetable questionnaire, *FFQ* food-frequency questionnaire, *N/A* not available

*p = 0.0471

^a^Excluding potatoes; ^b^Correlations were similar when they were also adjusted for age

**Fig. 2:**
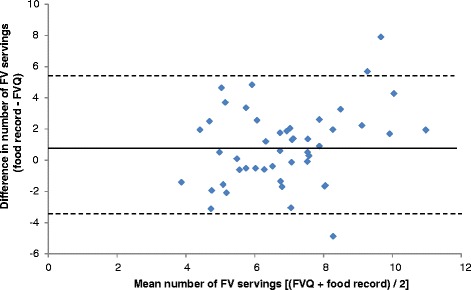
Bland-Altman plot showing agreement between the fruit and vegetable questionnaire and the food record. *Note*. FV: fruit and vegetable; FVQ: fruit and vegetable questionnaire; FR: food record. The line in the middle represents the mean difference in the number of fruit and vegetable portions between the two instruments (M = 0.91 ± 2.37) and the two other dotted lines represent the upper and lower limits of agreement (M ± 2SD = -3.83, 5.65)

According to the Bland-Altman plot showing the agreement between the FVQ and the food record, the mean difference in the number of servings reported between the FVQ and the food record was 0.91 ± 2.37 servings (see Fig. 2). This indicates that when pregnant women completed the food record, they usually reported consuming approximately one more serving of FV than when they answered the FVQ. Nevertheless, the majority of differences in servings between the two instruments were within two standard deviations of the mean difference.

### Test-retest reliability

The FVQ had overall a good test-retest reliability with good intra-class correlations between the two administrations of the questionnaire at a mean 28-day interval for all FV (range: 0.70–0.72), except for potatoes (ICC = 0.35) and vegetable juice (ICC = 0.24) whose intra-class correlations were mediocre according to Fermanian’s [37] classification. The intra-class correlations were: 0.71 for vegetable intake, 0.70 for fruit intake, 0.70 for fruit juice and for 0.72 FV intake.

## Discussion

Overall, the FVQ showed acceptable validity among our sample of pregnant women with significant correlations with a food record and a FFQ for all FV, except potatoes, even when controlling for energy intake and the presence of nausea. However, when pregnant women completed the FVQ, they tended to underreport the number of FV servings consumed compared to when they completed the food record. Also, none of the FV intake of the self-reported tools was significantly correlated to any of the plasma carotenoids measured in the present study, except for the FFQ and β-cryptoxanthin plasma concentrations when adjusted for the presence of nausea. The FVQ also has acceptable evidence of test-retest reliability with good intra-class correlations at a two-week interval, except for potatoes and vegetable juice.

The FVQ was moderately correlated to the food record and highly correlated to the FFQ. The correlation between FV intake measured by the FVQ and the FFQ (*r* = 0.61) was similar to the one that was found among obese (*r* = 0.66) and non-obese participants (*r* = 0.65) in Godin et al.’s [22] original study. The fact that the FVQ and the FFQ are both retrospective tools that measure frequency of consumption could partly explain why both instruments are more highly correlated compared with the food record which is a prospective measure. Retrospective measures are associated with memory biases, since participants have to recall what they ate over a specific period of time. In addition, since both the FVQ and the FFQ are both retrospective measures they likely also share dependant measurement errors and therefore a high correlation between the two tools does not necessarily mean that the FVQ has a high validity, but rather that the FVQ and its comparison method, the FFQ, assess FV intake similarly. Prospective measures, on the other hand, can lead to reactivity. Reactivity occurs when respondents change their diet to avoid the burden of recording foods, such as mixed dishes (i.e., recipes), or most importantly, when they adopt a more socially desirable diet [38]. Also, the order in which pregnant women had to complete the food record and the FVQ at the first mailing was not specified. If they completed the FVQ first this would mean that the FVQ (past 7 days) and the food record (prospective 3 days) would not cover the same period of time. In fact, this could explain why correlations between the FVQ and the FFQ are higher than with the food record. Ideally, the FVQ should have been completed first, since completing a food record could have led pregnant women to become more aware of their eating habits (including their FV intake), which could have influenced the responses they gave when filling out the FVQ.

Adjustments for energy intake and the presence of nausea did not change the correlations between the self-reported tools and with biomarkers, although approximately half of our sample reported experiencing nausea in the past month and close to 70 % of them mentioned that it affected their diet. This is contrary to a study by Brantsaeter et al. [39] which validated a FFQ against a food record in pregnant women and found that the correlation increased from 0.27 to 0.49 for energy and from 0.28 to 0.43 for protein when women who reported nausea were excluded. The same happened when their FFQ was validated with urinary nitrogen excretion; the correlation went from 0.27 to 0.58 for protein intake when participants with nausea were excluded [39]. However, it is possible that nausea affects dietary intake differently depending on type of food and maybe FV intake is less affected by nausea than protein intake in a similar fashion as taste and food preferences or aversions change across the course of pregnancy [40, 41]. Supplements use is also known to affect correlations between self-reported instruments, especially with biomarkers [42]. We did not adjust our correlations for this last variable given that all of our pregnant women, except two, reported using supplements, mainly prenatal vitamins with folic acid.

Results from this study suggest that the FVQ seems to underestimate FV intake compared to the food record. We cannot exclude the possibility that the food record may have overestimated FV intake rather than the FVQ underreporting FV intake. Indeed, given that food records imply writing down what is eaten at the time it is consumed, they are known to cause reactivity [38]. It is thus possible that pregnant women decided to eat more FV on the days they recorded their food intake to project a good image of themselves. In fact, the proportions of participating women who met Canada’s Food Guide recommendations for FV were higher (45.8 and 62.5 %) compared to another study in Canada which used a self-reported FFQ and reported that 35 % of pregnant women met the current Canadian recommendations [20].

Another possible explanation for a potential underestimation of FV intake is that the FVQ differs from the food record given that no specific instructions on mixed dishes of FV are provided. It is thus possible that pregnant women did not take into account mixed vegetable dishes (e.g., vegetable soup) or sauces (e.g., salsa) when they reported their FV intake using the FVQ. In fact, a review of brief survey instruments for the measurement of FV in adults found that the inclusion of questions on mixed vegetable dishes enhanced the validity of those questionnaires [43]. Authors interested in using the FVQ among pregnant women might want to add items on mixed dishes and verify if it enhances its validity compared to a food record.

None of the self-reported instruments were significantly correlated to any of the plasma carotenoids measured in the present study, even though we excluded potatoes from FV intake given their low content in carotenoids [44]. This could result from the period of time elapsed between the administration of the FVQ, the food record and the moment the blood sample was drawn (usually within two weeks after filling the FVQ and the food record). In fact, the only significant correlation was for the FFQ—which was administered during the same visit that the blood sample was drawn—and β-cryptoxanthin plasma concentrations when the correlation was adjusted for the experience of nausea. This last result is similar to two studies that found significant correlations between FV, excluding potatoes [45], and between FV [46], assessed by a FFQ and plasma β-cryptoxanthin concentrations in pregnant women. Similarly to our study, Brantsaeter et al. [45] reported non-significant correlations between FV intake, excluding potatoes, measured by their FFQ and α-carotene and sum of carotenoids while Vioque et al. [47] found non-significant correlations between FV assessed by their FFQ and plasma retinol and lycopene. Yet, some studies mentioned significant correlations between FV assessed by FFQ and certain plasma carotenoids, such as lutein [45, 47], β-carotene [47, 48] and α-carotene [47] and even with total carotenoids [47]. In sum, it is still unclear which carotenoids found in plasma are the most correlated to FV assessed by self-reported tools in pregnant women. It is possible that the present study lack statistical power to detect significant correlations between certain plasma carotenoids and FV because of low sample size compared to studies that found significant correlations. Other elements that could explain the poor correlations observed between plasma carotenoids and FV intake measured by the FVQ, the food record or the FFQ are within-subject variations in metabolism and carotenoids bioavailability [49]. Within-subject variations in metabolism can affect the absorption of plasma carotenoids while food preparation (e.g., adding oil or heating FV) can increase the bioavailability of their carotenoids content [49].

The test-retest reliability of the FVQ was mostly good, except for potatoes and vegetable juice. One problem with assessing test-retest reliability for questionnaires assessing eating behavior, such as the FVQ, compared to more stable attributes, such as personality traits or intellectual quotient, is that poor test-retest reliability scores can be due to natural variations in dietary intakes between the two assessment periods and not be a sign of low reproducibility. Unfortunately, it is not possible to statistically distinguish within-subject variations from random measurement errors [33]. Therefore, lower ICCs for potatoes and vegetable juice might be due to the fact that these foods are less commonly consumed compared to other categories of FV. In fact, they were the two categories of FV that had the lowest mean daily intakes in our sample. If these foods were consumed at the first administration of the FVQ and not at the second administration, or vice versa, this might lead to a lower ICC, since servings will differ between both time points. Nevertheless, when FV are combined, their ICC was good (0.72) and is similar to the study of Vian et al. [50] in which they administered a FFQ for consumption of polyphenol-rich foods in pregnant women at a 15 days interval and obtained an ICC of 0.73.

Strengths of this study were 1) the use of an objective measure of FV intake, plasma carotenoids, 2) the adjustment for the presence of nausea, which is specific to pregnancy and 3) the reporting of data on reproducibility, which is often lacking for brief FV survey instruments [43]. The main limitation of the study was the low sample size, even though pregnant women across all three trimesters were represented. Our sample was also comprised of female volunteers with high levels of education and high family income, which might limit generalizability to other populations of pregnant women.

## Conclusions

To conclude, the FVQ has acceptable validity and test-retest reliability values, but seems to underestimate FV servings in pregnant women. It represents an interesting alternative for researchers or clinicians interested in estimating quickly FV intake among pregnant women, such as in large trials or during prenatal visits. The FVQ should however be coupled with other self-reported measures, such as a food record, for assessing precise individual FV intake.

## Additional file

Additional file 1: The Fruit and Vegetable (FVQ) that was validated in the present study. (DOCX 20 kb)

## Footnotes

1 Since nausea is more usual in early pregnancy, adjustment for trimester was also computed. All correlations were similar to when they were adjusted for the presence of nausea (data not shown).
