# Supplementary material for: Validity and reliability of a brief self-reported questionnaire assessing fruit and vegetable consumption among pregnant women
Source: BMC Public Health. 2016 Sep 15;16:982. doi: 10.1186/s12889-016-3656-y (PMC5025575; doi:10.1186/s12889-016-3656-y)
Supplement: Additional file 1: — The Fruit and Vegetable (FVQ) that was validated in the present study. (DOCX 20 kb) [file 12889_2016_3656_MOESM1_ESM.docx]

| **Fruit and vegetable portion size definition** |
| --- |
| One portion of fruit or vegetable equals:  1 medium-size fruit or vegetable  ½ cup (125 ml) of cut fruit or vegetable  ½ cup (125 ml) of fruit juice  1 cup (250 ml) of green salad |
| **All these foods can be fresh, frozen or canned** |

1. **In the past seven days, how many servings of these foods did you eat?**

*Example: If you drank 250 ml of fruit juice during the two weekend days only, enter 0 in all weekday boxes and 2 in the Saturday and Sunday boxes.*

|  | **Monday** | **Tuesday** | **Wednesday** | **Thursday** | **Friday** | **Saturday** | **Sunday** |
| --- | --- | --- | --- | --- | --- | --- | --- |
| **Fruit juice** | 0 servings | 0 servings | 0 servings | 0 servings | 0 servings | 2 servings | 2 servings |

**Please indicate the appropriate number of servings in each box.**

|  | **Monday** | **Tuesday** | **Wednesday** | **Thursday** | **Friday** | **Saturday** | **Sunday** |
| --- | --- | --- | --- | --- | --- | --- | --- |
| **Fruit juice** | __ servings | __ servings | __ servings | __ servings | __ servings | __ servings | __ servings |
| **Vegetable juice** | __ servings | __ servings | __ servings | __ servings | __ servings | __ servings | __ servings |
| **Fruits** | __ servings | __ servings | __servings | __ servings | __ servings | __ servings | __ servings |
| **Potatoes (excluding French-fried potatoes)** | __ servings | __ servings | __ servings | __ servings | __ servings | __ servings | __ servings |
| **Green salads** | __servings | __ servings | __ servings | __ servings | __ servings | __ servings | __servings |
| **Other vegetables** | __ servings | __ servings | __ servings | __ servings | __ servings | __ servings | __ servings |

1. **Does your consumption of fruit and vegetable of the past 7 days corresponds to your eating habits of the past 3 months?**

🞎 Not at all representative of my eating habits

🞎 Not much representative of my eating habits

🞎 Sufficiently representative of my eating habits

🞎 Very representative of my eating habit

1. **How does your consumption of fruit and vegetable of the past 7 days differ from your eating habits of the past 3 months?**

**In the past 7 days, I consumed…**

🞎 Much more fruit and vegetable than in the past 3 months

🞎 A bit more fruit and vegetable than in the past 3 months

🞎 A bit less fruit and vegetable than in the past 3 months

🞎 Much more fruit and vegetable than in the past 3 months
